# Correlation between balance and the level of functional independence among elderly people

**DOI:** 10.1590/S1516-31802012000200005

**Published:** 2012-04-03

**Authors:** Melina Galetti Prata, Marcos Eduardo Scheicher

**Affiliations:** I Physiotherapist, Department of Physical Education, Universidade Estadual Paulista (Unesp), Rio Claro, São Paulo, Brazil.; II PhD. Physiotherapist and Assistant Professor, Department of Special Education, Universidade Estadual Paulista (Unesp), Marília, São Paulo, Brazil.

**Keywords:** Aging, Postural balance, Activities of daily living, Independent living, Movement, Envelhecimento, Equilíbrio postural, Atividades cotidianas, Vida independente, Movimento

## Abstract

**CONTEXT AND OBJECTIVE:**

As the overall number of elderly people increases there is a corresponding rise in the number of older persons with disabilities. In order to examine whether there is any relationship between balance and activities of daily living, we evaluated balance and daily activities among elderly people living in the community.

**DESIGN AND SETTING:**

Cross-sectional study at Universidade Estadual Paulista (Unesp), Marília, São Paulo, Brazil.

**METHODS:**

The study included 70 community-dwelling elderly people aged 65 and over. Balance was evaluated using the Berg Balance Scale (BBS) and physical independence in daily activities was evaluated using the Barthel Index (BI). The Spearman correlation coefficient was used to examine the relationship between the parameters evaluated. Significance was set at the level of 5% (P < 0.05).

**RESULTS:**

The population’s mean age was 70.5 ± 5 years. The mean BBS score was 50.9 ± 4.1, whereas the mean BI score was 98.1 ± 2.8. Statistically significant relationships were found between the BBS and BI (r = 0.41; P = 0.0004); between age and BI (r = -0.24; P = 0.04); and between age and BBS (r = -0.57; P = 0.0001).

**CONCLUSIONS:**

The results showed that among elderly people, there are correlations between age, balance and independence level.

## RESUMO

**CONTEXTO E OBJETIVOS:**

Como está ocorrendo um aumento do número total de pessoas idosas, há um aumento correspondente de idosos com incapacidades. Para examinar se há alguma relação entre equilíbrio e atividades de vida diária, nós avaliamos o equilíbrio e as atividades de vida diária em idosos da comunidade.

**TIPO DE ESTUDO E LOCAL:**

Estudo transversal na Universidade Estadual Paulista (Unesp), Marília, São Paulo, Brasil.

**MÉTODOS:**

O estudo incluiu 70 idosos moradores da comunidade com 65 anos ou mais de idade. O equilíbrio foi avaliado por meio da Escala de Equilíbrio de Berg (EEB) e a independência nas atividades de vida diária foi avaliada usando o Índice de Barthel (IB). O coeficiente de correlação de Spearman foi usado para examinar a relação entre os parâmetros avaliados. Foi fixado um valor significante ao nível de 5% (P < 0,05).

**RESULTADOS:**

A média de idade da população foi de 70,5 ± 5,0 anos. O escore médio da EEB foi de 50,9 ± 4,1, enquanto o do Índice Barthel foi de 98,1 ± 2,8. Uma correlação significativa foi encontrada entre os escores da EEB e o IB (r = 0,41; P = 0,0004), entre idade e Índice de Barthel (r = -0,24; P = 0,04) e entre idade e escores da EEB (r = -0,57; P = 0,0001).

**CONCLUSÕES:**

Os resultados mostraram que, em idosos, há correlação entre idade, equilíbrio e nível de independência.

## INTRODUCTION

The number of people over 60 years of age is projected to double over the next 20 years. Hence, reducing age-related disability is an essential public health goal. According to demographic projections, 33 million Brazilians will be older than 60 years of age in 2025.^1^

As the overall number of elderly people increases, there is a corresponding rise in the number of older persons with disabilities. Such disabilities may be social, physical, mental or psychological.^2^ Using data from the United States, it was estimated that 9.5 million non-institutionalized individuals experienced difficulty in performing basic activities such as walking, self-care and home-management activities.^3^ Of these, 59% were over the age of 65 years. In the 65-74-year-old age group, one in nine individuals had difficulty in performing basic activities. This ratio rose to one in four in the 75-84-year-old age group and three in five among individuals aged 85 years and over.

Declining physical function is associated with institutionalization, morbidity and mortality.^4^ Older people’s functional independence is an important indicator of their health status.

It is well known that loss of independence is one of elderly people’s greatest concerns. For this population, health is directly related to independence and the capacity to do things, work and come and go, even if the individual presents chronic diseases.^5^ Thus, the independence to perform activities of daily living becomes an essential element of proper development in old age and is part of the concept of successful aging.^6^^,^^7^

It has been suggested that elderly people present reduced ability to control their posture, which may predispose them to increased risk of falling.^8^ According to Jonsson,^9^ age-related deterioration of balance or postural control has a negative impact on the ability to safely carry out day-to-day activities. Studies among the elderly are particularly important, since this group is at greater risk of developing fractures and comorbidities associated with falls.

Among the likely causes of postural instability among the elderly, changes in the relationship between sensory information and motor action are of importance. The elderly have greater difficulty in interpreting sensory information and prioritizing it according to its relevance, and in selecting the proper response in order to maintain their balance in specific positions.^10^

Aging is associated with progressive loss of neuromuscular function that often leads to progressive disability and loss of independence.^11^ Sarcopenia and the characteristic skeletal muscle atrophy and weakness are considered to be major contributory factors towards the loss of functional mobility, independence, and frailty that is present in many older adults.^12^^,^^13^

## OBJECTIVE

The aim of the present study was to evaluate and correlate the balance, daily activities and age of elderly people living in the community.

## METHODS

### Subjects

A convenience sample of 70 community-dwelling elderly individuals was recruited at senior citizen centers in the city of Marília, São Paulo, Brazil.

The following were the inclusion criteria: age 65 years or older; residence in the community; and independent gait (without a gait assistance device). The following were the exclusion criteria: cognitive impairment detectable by the Mini-Mental State Examination (MMSE); and presence of factors that interfere in body balance, such as neuromusculoskeletal diseases (e.g. stroke or Parkinson’s disease), uncorrected visual problems, orthostatic hypotension, or continuous use of sedatives, antidepressants or hypnotics.

Written informed consent was obtained from all patients before enrollment. The study was submitted to and approved by the Research Ethics Committee of the University of Philosophy and Sciences, Universidade Estadual Paulista (Unesp), Marília, São Paulo, Brazil, and was carried out in accordance with Resolution no. 196/96 of the National Health Council.

### Study design

This was a cross-sectional study. Data were collected via face-to-face interviews by researchers. Demographic information within the scope of the present study, such as age, diseases and medications in use, was sought. The MMSE was administered to all participants. Every participant without cognitive impairment (i.e. MMSE ³ 18) was included in the study.^14^

### Outcome measurements: balance and independence

The participants’ balance was evaluated using the Berg Balance Scale (BBS), and functional independence in daily activities was evaluated using the Barthel Index (BI).

The BBS, which measures “functional balance”, has three dimensions: maintenance of a position, postural adjustment to voluntary movements and reaction to external disturbances. Subject performance in each of 14 activities is measured on a five-point ordinal scale ranging from 0 to 4 (0 = unable to perform; 4 = independent), such that the aggregate score ranges from 0 to 56. The average time taken to administer the scale was 10 to 15 minutes. Scores of 45 or less indicate inability to walk independently and safely in daily life.^15^

The BI, which measures independence in daily life, is composed of 10 items: feeding, bathing, grooming, dressing, bowel motion, bladder motion, toilet use, transfers to bed and chair and back, mobility and use of stairs. The score corresponds to the sum of all the points obtained, and can range from 0 to 100 points. Elderly people with scores from 0 to 20 are considered to be totally dependent; from 21 to 60, seriously dependent; from 61 to 90, moderately dependent; from 91 to 99, slightly dependent; and of 100, independent.^16^

### Data analysis

The statistical analyses were performed using the GraphPad Instat software. Values were recorded as mean ? standard deviation (SD). The Spearman correlation coefficient was used to examine the relationship between the evaluation parameters. Significance was set at the level of 5% (P < 0.05).

## RESULTS

Out of the 70 elderly individuals studied, 57 were women (81.4%) and 13 were men (16.6%), with a mean age of 70.5 ? 5.0 years.

Every participant had MMSE scores greater than 18 (25.5 ? 2.7). In the population studied, the mean number of medications taken was 2.5 ? 1.6 drugs/day. With regard to schooling level, nine participants (12.9%) were illiterate, 32 (45.7%) had attended school for 1- 4 years, 17 (24.3%) for 5-8 years and 12 (17.1%) for more than eight years.

The mean BI score for the women was 97.8 ? 2.9 and for the men, it was 99.6 ? 1.3 (P = 0.06), with an average of the group of 98.1 ? 2.8. The degree of balance was found to be lower among the women than among the men (50.6 ? 3.3 and 51.5 ? 4.3, respectively) (P = 0.96). The average score of the Berg Balance Scale for the studied group was 50.9 ? 4.1.

**Figure 1.**
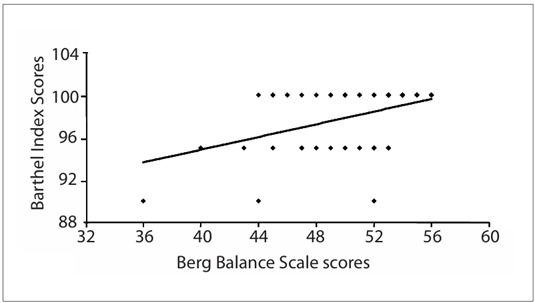
Relationship between Berg Balance Scale scores and Barthel Index scores among elderly people (n = 70); r = 0.41; P = 0.0004.

**Figure 2.**
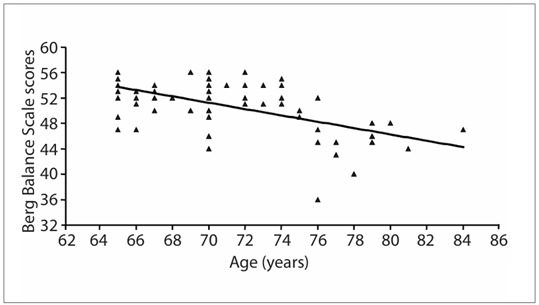
Relationship between age and Berg Balance Scale scores (n = 70); r = -0.57; P = 0.0001.

Correlation analysis on these data gathered from elderly people living in a community, showed that there was a statistically significant relationship between BBS and BI scores (r = 0.41; P = 0.0004), which was considered to be a moderate correlation (Figure 1). Figure 2 shows the association between the participants’ ages and their balance, as appraised using the BBS (r = -0.57; P = 0.0001). Correlations were also found between MMSE and balance (r = 0.42; P = 0.0003); between age and MMSE (r = 0.34; P = 0.0032); and between and BI (r = -0.24; P = 0.04). However, no statistically significant relationship was found between MMSE and BI (r = 0.05; P = 0.65).

Correlation analysis on the data also showed a statistically significant relationship between sex and BI (r = 0.33; P = 0.005). No statistically significant relationship was found between sex and BBS (P > 0.05). Another correlation analysis showed a statistically significant relationship between the number of medications taken and the BBS scores (r = -0.68; P < 0.0001). A correlation was also found between physical exercise practice and the BBS scores (r = 0.42; P = 0.0002), thus indicating that exercise practice can improve balance.

## DISCUSSION

Statistical projections have indicated that by 2050, elderly people will represent 16% of the Brazilian population. In absolute terms, these projections rank Brazil sixth in the world, in terms of elderly population, with more than 32 million elderly people.

In Brazil, between 1997 and 2007, the increase in the population in general amounted to 21.6%, against 47.8% for the group aged 60 years or older.^17^ The aim of the present study was to correlate age, balance and activities of daily living among community-dwelling elderly people.

The aging process is related to decreasing balance and ability to perform daily activities, and this situation may lead to falls, fear of falling, dependence, institutionalization and death. Specifically with regard to daily activities, the need for help to perform simple daily tasks such as eating, bathing and walking is associated with a large number of negative health indicators, such as hospitalization, treatment costs, quality of life and, finally, death.^18^ In our study, the participants presented good scores in the Barthel Index (98.1 ? 2.8), thus indicating only slight dependence in carrying out daily activities.

Dependence, by itself, does not constitute a negative event. At different stages of life, individuals may or may not be dependent, on either a temporary or a definitive basis. Dependence takes on greater importance when this appears because of events that occur during the final stage of life, and daily activities are affected by this dependence. According to Pires and Silva,^5^ loss of independence is one of elderly people’s greatest concerns. For this population, health is directly related to independence and the capacity to do things, work and come and go, even if these individuals present chronic diseases. If elderly people remain autonomous (with the capacity to choose and decide by themselves) and independent (with the capacity to carry out actions alone, without depending on others), the difficulties will be smaller, both for themselves and for their families and society.

Our results also showed that, among the elderly appraised here, there was an association between balance and daily activities, thus indicating that elderly individuals who had better balance kept a good level of independence. Mobility and functional level are among the most important factors necessary for an independent life. In this study, only nine participants (12.8%) had scores below 45 in the BBS. On the other hand, but with the same clinical meaning, other studies have shown that reduced balance may result in functional dependence among elderly people.^19^^,^^20^^,^^21^

This study is in agreement with the study by Yümin et al.,^22^ which found a correlation between balance (as appraised using the BBS) and activities of daily living (as appraised using the BI). In previous studies, no statistically significant relationship was found between functional balance (BBS) and the level of daily activities.^23^^,^^24^^,^^25^ Such differences may be due to the characteristics of the samples analyzed in these studies.

The physiological changes associated with aging affect the absorption, bioavailability, volume of distribution, metabolism and excretion of drugs. These effects apply to many of the medications commonly prescribed to older people, so it is not surprising that a link has been shown between several of these drugs and falls.^26^ Our results showed that there was a negative correlation between the number of drugs and balance, thus suggesting that there really is a relationship between larger numbers of medications in use and balance difficulties. However, it needs to be made clear that the strength of the association between drug use and falls among older adults is dependent on the class of medication in question, which was not appraised in this study.

Maintenance of balance and body posture in the standing position is essential for performing activities of daily living.^27^ In our study, the mean BBS score for the elderly individuals studied was 50.9 ? 4.1, with a range from 36 to 56 (with nine participants presenting scores less than or equal to 45). We believe that inclusion of balance training in care and rehabilitation programs for the elderly would be useful in assisting elderly people to maintain their functional independence. More comprehensive studies are needed on this subject.

## CONCLUSION

Among the community-dwelling elderly people who participated in this study, good balance and good performance regarding daily activities were found. Moreover, there was a relationship between balance and activities of daily living.
